# Induction of a rat model of premature ovarian insufficiency using D-galactose feeding during the critical periods of development: A pilot study

**DOI:** 10.18502/ijrm.v20i4.10904

**Published:** 2022-05-23

**Authors:** Marzieh Rostami Dovom, Mahsa Noroozzadeh, Nariman Mosaffa, Abbas Piryaei, Azita Zadevakili, Mohammad Amin Abdollahifar, Fahimeh Ramezani Tehrani

**Affiliations:** ^1^Reproductive Endocrinology Research Center, Research Institute for Endocrine Sciences, Shahid Beheshti University of Medical Sciences, Tehran, Iran.; ^2^Department of Immunology, School of Medicine, Shahid Beheshti University of Medical Sciences, Tehran, Iran.; ^3^Urogenital Stem Cell Research Center, Shahid Beheshti University of Medical Sciences, Tehran, Iran.; ^4^Department of Stem Cells and Developmental Biology, Cell Science Research Center, Royan Institute for Stem Cell Biology and Technology, ACECR, Tehran, Iran.; ^5^Department of Biology and Anatomical Sciences, School of Medicine, Shahid Beheshti University of Medical Sciences, Tehran, Iran.; ^6^Endocrine Research Center, Research Institute for Endocrine Sciences, Shahid Beheshti University of Medical Sciences, Tehran, Iran.

**Keywords:** Premature ovarian insufficiency, Animal model, D-galactose.

## Abstract

**Background:**

Premature ovarian insufficiency (POI) affects about 1% of women of reproductive ages (15-45 yr), with no curative treatment.

**Objective:**

We aimed to present a rat model of POI using a D-galactose enriched diet.

**Materials and Methods:**

In a pilot study, 4 pregnant Wistar rats were divided into 4 groups; 3 groups were fed galactose-enriched diets at days 3-15 of pregnancy (G1); on the 3 ${}^{\text{rd}}$ day of pregnancy to parturition (G2), and the 3 ${}^{\text{rd}}$ day of pregnancy until the end of the weaning period (G3). Also, group 4, as the control group (G0), was fed standard pellets during the study. After confirming the lack of adverse effects of dieting with galactose in terms of offsprings' birth weight, we performed our study designed the same as the pilot study. A total of 40 pregnant Wistar rats were randomly divided into 4 groups. Ovarian histology, reproductive hormones, and immunological characteristics of the female offspring were examined in all experimental groups and compared.

**Results:**

The pilot study revealed no significant differences in the birth weight of the offspring of the 4 study groups (p = 0.96). The ovarian index in the female offspring of those with a gal-exposed diet was significantly lower than that of the control group offspring (p < 0.01).

**Conclusion:**

As the birth weights of the offspring of our experimental and control groups were similar, it can be concluded that the reduction of ovarian follicles after prenatal exposure to D-galactose is due to the ovotoxicity of galactose. The results of our final study will provide more information about the rat POI model induced by prenatal exposure to D-galactose.

## 1. Introduction

Premature ovarian insufficiency (POI) is characterized by oligomenorrhea or absent menstrual periods, elevated follicle-stimulating hormone (FSH) levels > 40 mIU/mL and decreased estrogen levels in women below 40 yr of age, and it is a critical condition for women of reproductive age (15-45 yr) (1). The prevalence of POI is about 1-5.5% in the general population (2, 3). The non-protective effects of estrogens on the cardiovascular system and bones in affected women may lead to many health problems, such as infertility, osteoporosis, cardiovascular diseases, and metabolic disorders, and decreased quality of life (4-6). So far, no treatment has been developed for POI, and management strategies only include symptomatic and maintenance therapies (1). Several animal models have been designed to understand the mechanisms of the disease; however, an optimal model has not been developed yet (7-9).

Galactosemia is an autosomal recessive disorder that causes the accumulation of galactose (C ${}_{6}$ H ${}_{12}$ O ${}_{6}$ ) and its toxic metabolites in the body due to an enzymatic defect in the conversion of galactose to glucose (10). The ovotoxic effect of galactose (as the main monosaccharide of the production of dairy such as milk, yogurt, and cheese) and its metabolites are well documented in galactosemic patients (11, 12), and POI is one of the common conditions in women affected by galactosemia (13). Some studies have used galactose to induce POI in laboratory animals (14, 15). Exposure to galactose during the perinatal period causes the destruction and reduction of small ovarian follicles (primordial and primary), while exposure to galactose after birth results in the destruction of larger growing follicles (preantral and antral) (15).

Prenatal galactose exposure appears to be a more successful model of POI than postnatal interventions, especially due to the reversible ovotoxic effects of galactose during the postnatal period (16). According to previous studies, gestational exposure of pregnant rats to 35% galactose feeding on day 3 of conception reduced the number of small ovarian follicles in the female offspring. On the other hand, some other studies have shown acceleration of apoptosis in follicles, which suggests the high acceleration of follicle apoptosis (17).

Regarding the time- and dose-dependent ovotoxicity of galactose, in the present study we aimed to produce a rat model of POI in the female offspring following maternal exposure to a galactose-enriched diet during gestation and weaning. We also aimed to examine the offspring of all experimental groups in terms of ovarian macroscopic and microscopic morphologies, reproductive hormones, and immunological and metabolic characteristics at postnatal days (Paroxysmal nocturnal dyspnea [PND] 45, 105, and 180 of age) and to compare them with the controls.

## 2. Materials and Methods

To develop a final study protocol, a pilot study was conducted from April 2019 to November 2019 among 4 groups, including 3 experimental and one control group at Reproductive Endocrinology Research Center, Research Institute for Endocrine and Metabolism, Shahid Beheshti University of Medical Sciences, Tehran, Iran.

### Pilot study

#### Animals

4 adult female Wistar rats (160-180 gr) were obtained from the animal house of Research Institute for Endocrine and Metabolism and maintained under standard husbandry conditions in a standard 12 hr light/dark cycle (with lights on at 06:00 daily) with controlled temperature (22 ± 3 C) and a relative humidity of 45-55%. Rats were divided into 4 groups. Animals had access to food and waterad libitum. In the first step of the study, each female rat was assigned to each group and mated with a young fertile male overnight in a separate polypropylene cage (43 cm × 30 cm × 15 cm). The first day of pregnancy was confirmed by observing a vaginal plaque or sperm existence in the vaginal smear.

#### Food supplementation 

The standard food pellets were purchased from Pars Co. Iran, and enriched with D-galactose (Souvenir Chemicals, Mumbai, India) in the animal house for further experimental groups. To prepare one kilogram of galactose-enriched food, 650 gr of standard food powder was mixed with 350 gr of galactose powder in distilled water. After converting to pellets and drying, the prepared food was stored in a refrigerator (2-4ºC) for further use.

#### Protocol for pilot study

**Figure 1:**
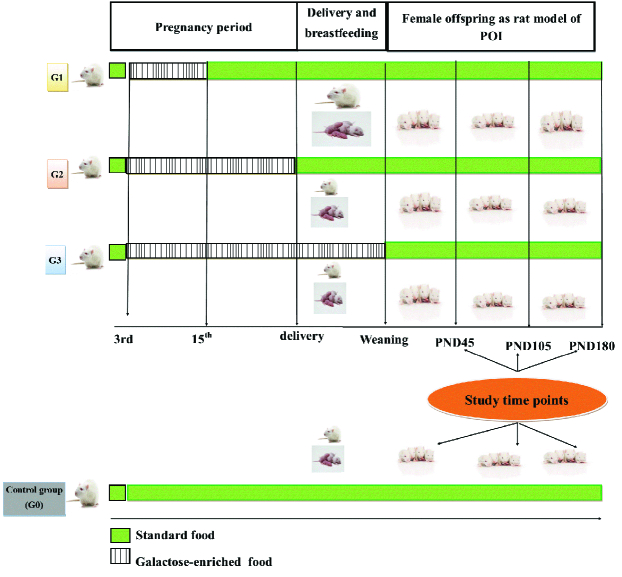
Schematic view of study design. POI: Premature ovarian insufficiency, PND: Paroxysmal nocturnal dyspnea.

After confirmation of pregnancy, female rats were kept in separate cages and divided into 4 groups the control group (G0) received only standard pellets during the whole time of the study. Group 1 (G1) received a galactose-enriched diet during the 3 ${}^{\text{rd}}$ -15 ${}^{\text{th}}$ days of pregnancy and continued with standard pellets until weaning. Group 2 (G2) received a galactose-enriched diet from the 3 ${}^{\text{rd}}$ day of pregnancy until parturition and continued with a standard pellets. Group 3 (G3) received galactose enriched pellets from the 3 ${}^{\text{rd}}$ day of conception until weaning. After delivery, the male offspring of each group were released from the cage, and the birth weight of the female pups of each group was recorded. At PND 45, the bodyweight of the pups in each group was recorded, then their ovaries were removed under deep anesthesia using an intraperitoneal injection of 5 gr of pentobarbital sodium. Ovarian weight was recorded, and the ovarian index was calculated by dividing ovarian weight by body weight. A schematic summary of the study design can be observed in figure 1.

### Development of final study protocol

#### Step 1: Induction of POI model

40 Wistar rats were randomly assigned to 4 groups (n= 10/ group). To minimize variations between the groups, random allocation was conducted using a previously described block randomization method. The groups were the same as described in the pilot study and received the diets explained previously.

#### Step 2: Model assessment in the female offspring

The body weight and the number of female offspring were recorded in each group. The vaginal opening time, regularity of the estrous cycle, number of nipples, vaginal length, anogenital distance, and anovaginal distance were recorded for the female offspring in all study groups.

#### Step 3: Blood collection and sampling of ovaries 

**Figure 2:**
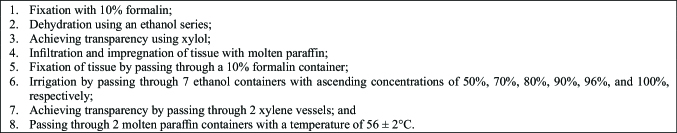
The automatic process in the automatic tissue processor machine.

Blood and ovarian samples were collected during the estrous phase of the sexual cycle at 3 time points, that is, PND 45, 105, and 180 of age; the day of birth was considered as PND one. Sampling was carried out at 8-9 AM after confirming the estrous phase by preparing a vaginal smear. The rats were then anesthetized using an intraperitoneal injection of 5 gr of pentobarbital sodium (60 mg/kg body weight; P3761; Sigma, St. Louis, MO, USA). The blood samples were directly collected from the abdominal aorta after centrifuging at 6000 cycles/mint for 5 min at 4 C. The sera were then separated and stored at -80 C for subsequent measurements of FSH, estradiol (E2), and Anti-Müllerian hormone (AMH) levels. Next, the ovaries were removed, trimmed excess fat, and fixed in 4% paraformaldehyde at room temperature for 3 days. They were then processed, based on the standard tissue processing protocol, and embedded in paraffin. The histological assessment of ovarian tissues was carried out after tissue preparation. This process includedfixation*, *in whichthe right ovaries of rats were placed in an automatic tissue processor machine for 24 hr. This machine automatically made the necessary changes to tissues during the following steps (Figure 2).

A paraffin block was individually prepared from each ovary. To quantify the histological parameters, distinct sections (10 µm) with 10-section intervals were mounted onto microscope slides to be stained with hematoxylin and eosin and labeled.

### Laboratory material

The enzyme-linked immunosorbent assay (ELISA) is a widely used tool for detecting and quantifying proteins and antigens from various samples. The AMH, E2, and FSH levels were measured using an ELISA assay based on the manufacturer's protocol. The following materials were commercially available in this study: phenobarbital sodium (5 gr; P3761; Sigma, St. Louis, MO, USA); Rat AMH ELISA Kit (Lot ZB-MI19822; Zellbio, Germany); Rat FSH ELISA Kit (Cat. No.: ZB-10182C-R9648; Zellbio, Germany), E2 ELISA Kit (Cat. No.: 4925-300A; Monobind, USA), and pentobarbital sodium (5 gr; P3761; Sigma, St. Louis, MO, USA). The serum glucose and insulin levels were measured by the glucose oxidase method (Pars Azmoon Co., Tehran, Iran) and the ultrasensitive rat insulin ELISA assay (Mercodia, Sweden). The serum levels of triglyceride, total cholesterol, and high-density lipoprotein were measured by enzymatic colorimetric (Pars Azmoon Co., Tehran, Iran) and enzymatic photometric methods (Randox, UK), according to the manufacturer's protocols.

### Morphological classification of follicles

The follicles were categorized based on a classification described by Myers et al. (18). Briefly, in this classification, a primordial follicle refers to a follicle with an oocyte surrounded by a single layer of flattened squamous granulosa cells. Occasional follicles, as the boundary between primordial and primary follicles, have both cuboidal and squamous cells. These follicles are considered primary follicles if the number of cuboidal cells is predominant. A primary follicle represents a follicle with an oocyte, surrounded by a single layer of cuboidal granulosa cells. Meanwhile, secondary follicles refer to follicles with more than one layer of cuboidal cells with no visible antrum. Finally, early antral follicles represent one or 2 small areas of follicular fluid (antrum), and antral follicles represent follicles with a large antrum.

### Determination of ovarian volume

**Figure 3:**
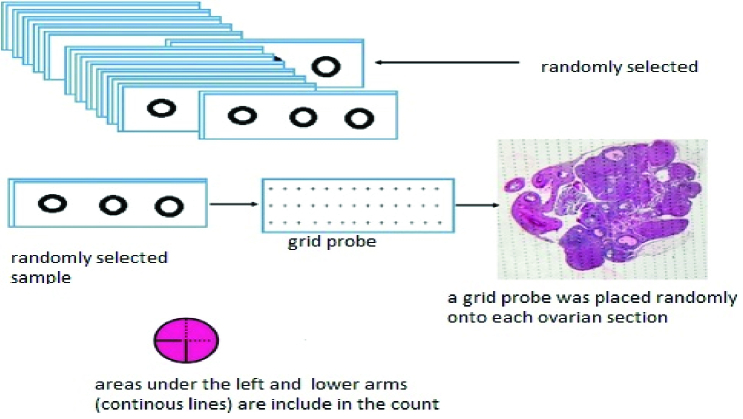
Schematic of how to select a sample and the process of estimating ovarian volume by the Cavalieri method.

In this study, the Cavalieri segmentation method with the point-counting technique, as a systematic random sampling method, was used to estimate the ovarian volume (19). For this purpose, a set of Cavalieri sections was prepared. For each ovary, 10 sections were sampled with a fractionator. A grid probe was placed randomly onto each ovarian section (Figure 3). Next, the number of points hitting the region of interest was counted. The whole ovarian tissue section was cut and counted for measuring the sample size. Finally, the ovarian volume was calculated using the following formula: 


V=∑p*a/p*t

Where *V* represents volume; *p* represents the points counted on the grid; *a/p* denotes the area associated with a point (0.02 mm in this study), and *t* denotes the section evaluation interval.

### Determination of anti-ovarian antibodies (AOAs)

The group with the highest similarity to POI was considered the optimal group (POI+). To determine AOA, the ovaries of rats were removed in the experimental and healthy control groups under deep anesthesia. They were then rinsed thoroughly with phosphate-buffered saline (PBS) (DNA biotech cat No. DB0017, Iran) as a cold medium and then divided into segments of 1 × 1 mm^2^. Next, the ovarian segments were homogenized in a tissue grinder. The grilled content was passed through a mesh nylon filter (40 µm) and centrifuged at 6000 cycles/mint for 20 min at 4ºC. After centrifugation, the supernatant was removed and passed through a filter (0.22 µm) to keep the liquid sterile.

Afterward, the protein concentration was determined by a Bradford assay. After determining the protein concentration, it was used as an antigen to cover the 96-well ELISA plate bed. The serum of rats was considered a possible source of AOAs and was examined by serial dilution. By creating serial dilutions of the studied sera, we increased the accuracy and sensitivity of the ELISA assay, which is important for determining sera with positive reactions to ovarian antigens and eliminating serum cross-reactivity.

In the next stages of the ELISA assay, the ELISA plate bed was covered with 0.5-5 μg of antigen, the protein extracted from the ovaries of healthy rats. The coated plates were then incubated with antigens in a refrigerator overnight. After removing the antigens from the refrigerator the following morning, the plates were allowed to reach room temperature and washed 3 times with PBS solution. Next, dilutions of 1.10 of each serum sample were added to the antigen, and incubation was performed at room temperature for 2 hr. The reacting sera were removed and washed again with PBS 3 times.

By adding a peroxidase-conjugated antibody against the rat antibody, the main reaction was induced. Incubation was performed for 1 hr at room temperature to identify antibodies bound to the ovarian antigen. The contents of the wells were re-harvested and washed with PBS 3 times. Following that, the reaction substrate (tri-methylbenzene) was added and incubated for 30 min in the dark at room temperature. Then, normal sulfuric acid (1 N) was added to terminate the substrate enzyme reaction. Finally, readings were obtained using an ELISA spectrophotometer over a range of wavelengths (450 nanometer wavelengths), and the optical density was recorded. Also, comparisons were made with the cut-off level of reacting serum in healthy rats.

### Measurement of body weight, puberty, and estrus cycle 

The female offspring of each mother was weighed, and its weight was recorded at PND one and every 15 days until the end of experiments. The vaginal opening day was considered a sign of puberty. The estrous cycle was monitored by daily observation of vaginal smears in allfemale offspring at 70-80 days of age (between 08.00 AM and 12.00 PM) for 10 consecutive days. To collect vaginal samples, the lips of the vulva were parted, and a cotton-tipped sterile swab was inserted into the vagina. The swab was rotated 2 or 3 times against the vaginal wall and then withdrawn and rolled on a clean glass slide. The smears were fixed with 70% ethanol, stained with Giemsa, and examined under a light microscope (x100 magnification). The proportions of leukocytes, epithelial cells, and cornified cells were determined in daily vaginal smears. Overall, these proportions change characteristically in different stages of the estrous cycle. The rat estrous cycle (proestrus, estrus, metestrus, and diestrus) lasts 4 days (20).

### Selection of the optimal model

The histological features of the ovaries, as well as the serum levels of some reproductive hormones (FSH, AMH, and E2) of the female offspring, were compared at 3-time points (PND 45, 105, and 180 of age) between the experimental and control groups. The optimal rat model of POI included rats with the lowest number of primordial and primary follicles, the lowest level of AMH, and the highest level of FSH in the blood serum, compared to the other experimental and control groups.

### Ethical considerations

This experimental study was approved by the Ethics Committee of the Endocrine Sciences Research Institute of Shahid Beheshti University of Medical Sciences, Tehran, Iran (Code: IR.SBMU.ENDOCRINE.REC.1398.001).

### Statistical analysis

The SPSS Statistics for Windows, version 20 (SPSS Inc., Chicago, Ill., USA) and STATA version 13 (STATA Inc., College Station, TX, USA) were used to analyze the findings. The comparison of the findings of the pilot study were analyzed by the Kruskal-Wallis non-parametric test. In the descriptive information section, numerical variables are presented as median (interquartile rate), and p < 0.05 was considered as the significance level.

## 3. Results

**Figure 4:**
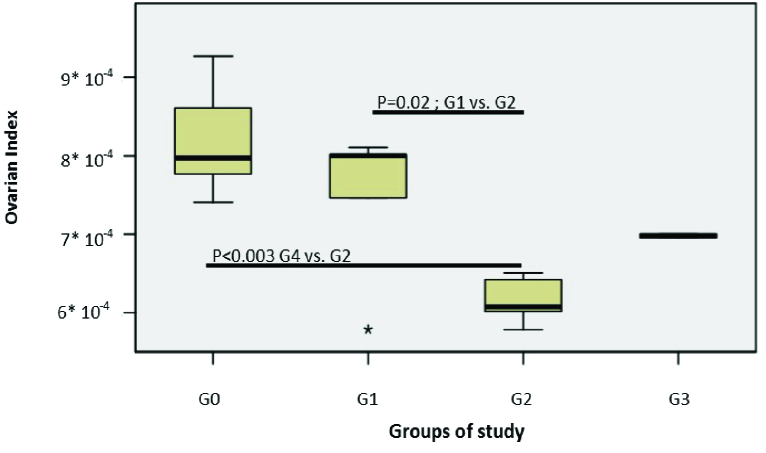
Comparison of ovarian index among study groups at PND 45 in the pilot study*.* Ovarian index: Ovarian weight to body weight ratio, G0: Control group, G1: Including pregnant rats fed 35% galactose diet from the 3 ${}^{\text{rd}}$ -15 ${}^{\text{th}}$ day of conception, G2: Including pregnant rats fed 35% galactose diet from the 3 ${}^{\text{rd}}$ day to parturition, G3: Including pregnant rats fed 35% galactose diet from the 3 ${}^{rd}$ day to weaning.

The birth weight was assessed in the different groups, and the ovarian weight and ovarian index. The results showed no statistically significant differences in birth weight between the galactose exposed experimental groups compared to the control group (p = 0.96). The comparison of the ovarian index at PND 45 showed a significantly lower ovarian index in those prenatally exposed to galactose vs. the control group (p < 0.01) (Figure 4).

## 4. Discussion

In this experimental study, we aimed to propose a rat model of POI using a galactose-enriched diet and to investigate changes in the ovarian follicles and some reproductive hormones. Since a galactose-enriched diet can be toxic to the reproductive system, especially the ovaries, it can disrupt the folliculogenesis process; therefore, it can be potentially used to create the closest model of POI. The lack of observing significant differences in the birth weight of first-generation gal-exposed female offspring compared to the control group suggested that the possible differences observed in ovarian follicles between the different groups were only related to the toxicity of galactose. The lower ovarian indexes also supported the hypothesis of galactose ovotoxicity.

Some studies have shown that reduced nutritional intake during the fetal period leading to destructive changes in the primordial follicles, reduces the ovarian reserves in the female fetus (21, 22). In the present study, a lack of observing such effects on the body weights of our study groups revealed that the addition of 35% galactose to the standard diet of rats did not impair general fetal growth; therefore, differences observed in the ovarian index were likely due to the toxic effect of galactose on the ovaries. Furthermore the weight loss of the ovaries under exposure to toxic substances indirectly confirmed their effect on the ovaries (23). Our results of the pilot study are in agreement with other studies that have reported a reduction in the ovarian index after exposure to galactose (24-26).

Galactose is a monosaccharide that is naturally produced and enters the body through nutrition (dairy products) (27). Almost all breastfed newborns are exposed to this monosaccharide from birth and even earlier in the mother's body. However, only high levels of this monosaccharide have toxic effects on the ovaries, as its metabolism is beyond the animals' tolerance (28). People with galactosemia, who are genetically deficient in galactose-metabolizing enzymes, are exposed to the toxicity of galactose aggregation or its metabolites. One of the target tissues of this toxicity is ovarian tissue, and women with galactosemia will suffer from POI if they survive (24); the use of galactose to induce POI was based on this background knowledge. According to previous studies, exposure to a galactose-enriched diet (35%) is suitable for inducing a rat model of POI (15, 29). However, there are very limited studies on the proper time of POI initiation (15, 7), and no study has yet examined the associated long-term changes in the ovaries.

Ovarian reserve is a non-renewable pool of primordial follicles that are formed by the process of growth and differentiation of primary germ cells from the yolk sac, and they migrate to the primary gonadal ridge (around the 5 ${}^{\text{th}}$ wk of the embryonic period) (30). In humans, around puberty the quiescent primordial follicles, under the influence of genes and signals from the oocytes, are recruited from the pool of ovarian reserves and go through their developmental stages (31). Under the influence of FSH, secreted by the anterior pituitary gland, primordial follicles begin develop. Structural changes in the pre-granulosa cells that surround oocytes and their transformation into granulosa cells in interaction with theca cells, derived from ovarian stromal tissue, activate the secretion of androgen and estrogen hormones (32, 33). Under any disruption of hormone secretion or follicular cells, the growth of follicles is impaired. Folliculogenesis is a process that begins in the fetal period; therefore, a model of POI must be induced during fetal life, especially in POI with low ovarian reserves from birth. The earliest studies using oral galactose have shown that a suitable dose of this monosaccharide (35%) has relatively low mortality in animals, despite its association with ovarian toxicity (29). Ovotoxicity of galactose has been shown to affect the growth of ovarian follicles and disrupt the migration of germ cells from the yolk sac to the gonads, resulting in low ovarian reserve. The mechanism of this process has been attributed to defects in some growth factors, such as growth differentiation factor 9, but its main mechanism has not been fully elucidated (33). The AMH level is an appropriate indicator of follicular reserves (primordial follicles), although it does not indicate the number of follicles. Lack of growth of ovarian follicles prevents the secretion of an adequate amount of E2, and the negative feedback process on the anterior pituitary gland is disrupted, which leads to an increase in FSH and decreased E2 levels in the sera (32).

In this study, we evaluated the prenatal and/or early postnatal exposure to galactose and examined the ovarian characteristics of the female offspring to develop a POI model. The number of ovarian follicles was examined by ovarian histopathology and AMH changes were investigated as a suitable indicator of ovarian reserves. The clinical symptoms of POI are amenorrhea and/or oligomenorrhea, increased FSH, and decreased estrogen. In this study, the FSH and E2 levels were also investigated. However, changes in puberty and examination of the estrous cycle can also show a good picture of changes in the hypothalamic-pituitary-ovarian axis.

Other methods for developing animal models of POI have some limitations, especially in the case of exposure to chemotherapy drugs. Although not impossible, it is very difficult to design an animal model of the fetal period, given the high toxicity of some chemical agents (34). Also, other methods, such as radiotherapy, have similar limitations. In methods such as ovarian removal (oophorectomy), significant information about the ovarian processes is lost. Therefore, it is more favorable to use a method associated with minimum mortality to mimic POI during critical periods of development.

In this study, we investigated both short- and long-term effects of prenatal and early postnatal exposure of pregnant rats to enriched galactose feeding to propose a suitable model for one of the common POI phenotypes (i.e., gradual reduction of ovarian follicles) in the female rat offspring. We also studied the microscopic and macroscopic morphological changes in the ovaries and investigated hormonal, metabolic, and immunological changes in the experimental groups vs. the controls.

## 5. Conclusion

As the birth weights of our experimental and control groups were similar, it seems that the lower levels of ovarian follicles after prenatal exposure to D-galactose was likely due to the ovo-toxicity of galactose. The results of our final study will provide more evidence in terms of the appropriateness of the rat POI model induced by prenatal exposure to D-galactose by reporting a fuller picture of declines in ovarian follicles along with hormonal changes.

##  Conflict of Interest

The authors declare that there is no conflict of interest.
